# Discovering Geographical Flock Patterns of CO_2_ Emissions in China Using Trajectory Mining Techniques

**DOI:** 10.3390/ijerph20054265

**Published:** 2023-02-27

**Authors:** Pengdong Zhang, Lizhi Miao, Fei Wang, Xinting Li

**Affiliations:** 1 School of Geographic and Biologic Information, Nanjing University of Posts and Telecommunications, Wenyuan Road 9, Nanjing 210023, China; 2 Smart Health Big Data Analysis and Location Services Engineering Research Center of Jiangsu Province, Nanjing University of Posts and Telecommunications, Wenyuan Road 9, Nanjing 210023, China; 3 East China Sea Fisheries Research Institute, Chinese Academy of Fishery Sciences, Shanghai 200090, China

**Keywords:** carbon emission, geographical flock pattern mining, attribute trajectory, bivariate time series, climate change

## Abstract

Carbon dioxide (CO_2_) emissions are considered a significant factor that results in climate change. To better support the formulation of effective policies to reduce CO_2_ emissions, specific types of important emission patterns need to be considered. Motivated by the flock pattern that exists in the domain of moving object trajectories, this paper extends this concept to a geographical flock pattern and aims to discover such patterns that might exist in CO_2_ emission data. To achieve this, a spatiotemporal graph (STG)-based approach is proposed. Three main parts are involved in the proposed approach: generating attribute trajectories from CO_2_ emission data, generating STGs from attribute trajectories, and discovering specific types of geographical flock patterns. Generally, eight different types of geographical flock patterns are derived based on two criteria, i.e., the high–low attribute values criterion and the extreme number–duration values criterion. A case study is conducted based on the CO_2_ emission data in China on two levels: the province level and the geographical region level. The results demonstrate the effectiveness of the proposed approach in discovering geographical flock patterns of CO_2_ emissions and provide potential suggestions and insights to assist policy making and the coordinated control of carbon emissions.

## 1. Introduction

Climate change is one of the biggest challenges faced by humankind, and its impact is becoming increasingly significant [1,2]. It can greatly affect the environment, production system, survival and development of humankind [3,4,5], hence typical extreme events and disasters (e.g., storms, droughts, heatwaves, fires, and floods) have become stronger and more frequent [6]. The 2022 Intergovernmental Panel on Climate Change (IPCC) annual report demonstrates that the world faces unavoidable climate hazards over the next two decades with global warming of 1.5 °C (2.7 °F), and urgent actions are required to deal with increasing risks [7]. One commonly acceptable solution is to make rapid and deep cuts in greenhouse gas emissions, particularly carbon dioxide (CO_2_) [8].

The majority of countries in the world have been seeking effective ways to reduce CO_2_ emissions. According to previous research, China is currently the largest emitter of CO_2_, accounting for 31% of global CO_2_ emissions in 2020 due to its rapid economic development and urbanization [9]. To achieve the net-zero or near-zero CO_2_ emissions, the Chinese government has launched a long-term mitigation goal, which indicates that China would reach peak emissions by 2030, and achieve carbon neutrality by 2060 [10]. Motivated by this national strategy, emerging research regarding carbon emissions in China has been paid much attention in various domains. Typical research focuses include the modelling, prediction, trading and policy evaluation of carbon emissions [11,12,13,14,15,16]. The establishment of effective policies to reduce carbon emissions is critical. Nevertheless, the long-term reduction of carbon emissions remains a key policy challenge for China and the world [17]. To meet the requirements to establish effective policies, specific, precise, and flexible policies must be proposed to different levels of geographical units (e.g., county, city, province, region, and country). Understanding the trends and trajectories of carbon emissions remains challenging in light of uncertainty about world economies and technological breakthroughs [18]. In this regard, the discovery of potentially important emission patterns must be performed from historical carbon emission data. The discovery of emission patterns plays a significant role in guiding the formulation of specific, precise, and flexible policies and the coordinated control of carbon emissions. Therefore, this paper focuses mainly on discovering important patterns from carbon emission data.

Carbon emission data are usually time series data, and time series can be classified into three types, i.e., univariate time series, bivariate time series, and multivariate time series [19,20]. In this paper, we are particularly interested in bivariate time series, because they can provide more abundant information than conventional univariate time series; furthermore, related consumptions (e.g., time) can be saved compared with complex multivariate time series. As a bivariate time series integrates the information of two different attributes into one coordinate system, it can be fully transferred to an attribute trajectory, which was introduced in [21] as a novel kind of trajectory. On this basis, traditional trajectory mining techniques can thus be adopted to discover desired movement related patterns.

Nowadays trajectory data are ubiquitous, having benefited from the proliferation of location aware techniques such as the global navigation satellite system (GNSS), Bluetooth, radio frequency identification (RFID), and Wi-Fi. The discovery of movement patterns has an important place in the domains of trajectory data mining as these patterns can exhibit the rules of individuals’ movements and their interactions. Typical movement patterns include the flock pattern [22,23,24,25,26], convoy pattern [27,28,29], leadership pattern [30,31,32], moving cluster [33,34], and crew [35]. Among these, the flock pattern has been paid much attention and it commonly plays an important role in many application fields. A flock can be informally depicted as “a group of spatially close objects staying together for a specific time duration”. Correspondingly, assuming geographical units are considered as objects and their attributes staying close for a specific time duration, these geographical units can be regarded as forming a flock. This way, the traditional flock existing between moving objects can be extended to geographical flock existing between geographical units. Given the importance of discovering flock patterns in traditional trajectory data and the shortage of flock pattern discoveries from new attribute trajectory data, this paper aims to discover geographical flock patterns from carbon emission data.

**Figure 1:**
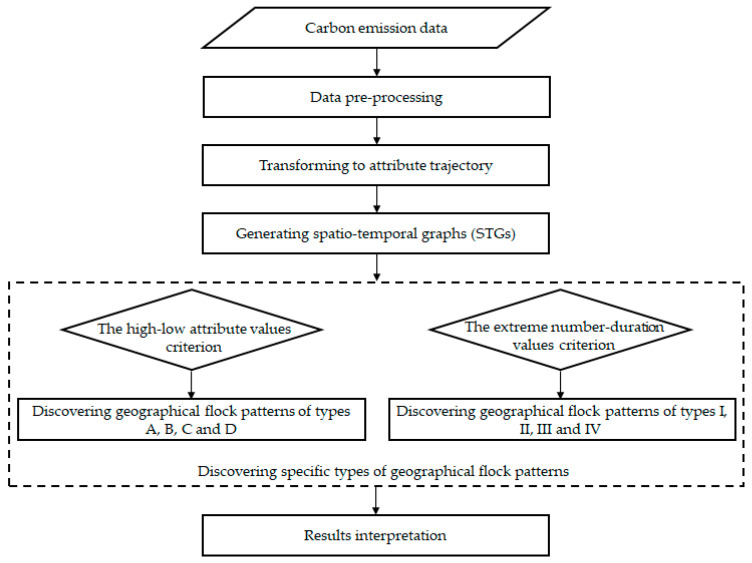
The framework of this study.

To achieve the discovery of geographical flock patterns, we propose a spatiotemporal graph (STG)-based approach. The proposed approach includes three main parts: first, the carbon emission data are transformed to attribute trajectory data; second, the STGs are generated from attribute trajectory data; third, specific types of geographical flock patterns are discovered from STGs. We adopt two criteria (i.e., the high–low attribute values criterion, and the extreme number–duration values criterion) to derive various types of geographical flock patterns. In short, four corresponding but different types of geographical flock patterns can be derived according to each criterion. A case study is conducted to verify the usefulness and applicability of the proposed approach, in which the original province-level CO_2_ emission data in China are employed. The geographical flock patterns of CO_2_ emissions are discovered at two levels, i.e., the province level and the geographical region level. The findings of this study may provide us with potential suggestions to assist policy making and the coordinated control of carbon emissions. The framework of this study is shown in Figure 1. According to this framework, important patterns (i.e., the geographical flock patterns in this paper) can be discovered from carbon emission data, and effective policies in reducing CO_2_ emissions may thus be formulated with the support of the discovered important patterns.

## 2. Materials and Methods

### 2.1. Basic Concepts

To facilitate understanding, we introduce three basic concepts in the following, namely bivariate time series, attribute trajectory and geographical flock.

#### 2.1.1. Bivariate Time Series

**Definition** **1** **(Time** **series).** *Given an object O, assume the value of a specific attribute of O at timestamp* $t_{i}$ *is* $v_{i}$ *(where* 1≤i≤n *), then the continuous segments of* $TS=\left\{\left(v_{1},t_{1}\right),\left(v_{2},t_{2}\right),\ldots ,\left(v_{n},t_{n}\right)\right\}$ *is considered as a time series.*

**Definition** **2** **(Bivariate** **time** **series).** *Given an object O, assume the values of two specific attributes of O at timestamp* $t_{i}$ *are* $u_{i}$ *and (where* 1≤i≤n *), respectively, then the two continuous segments of* $TS_{1}=\left\{\left(u_{1},t_{1}\right),\left(u_{2},t_{2}\right),\ldots ,\left(u_{n},t_{n}\right)\right\}$ *and* $TS_{2}=\left\{\left(v_{1},t_{1}\right),\left(v_{2},t_{2}\right),\ldots ,\left(v_{n},t_{n}\right)\right\}$ *in the same coordinate system is considered as consisting of a bivariate time series.*

#### 2.1.2. Attribute Trajectory

**Definition** **3** **(Trajectory).** *Given a moving object O, assume its spatial location at* $t_{i}$ *during the movement path are* $\left(x_{i},y_{i}\right)$ *(where* 1≤i≤n *), then* $Traj=\{\left(x_{1},y_{1},t_{1}\right),\left(x_{2},y_{2},t_{2}\right),\ldots ,\left(x_{n},y_{n},t_{n}\right)$ *is considered as a trajectory.*

Note that in Definition 3, (*x*, *y*) denotes the spatial location (e.g., latitude and longitude) of a moving object. In addition to spatial location, (*x*, *y*) can denote other attributes as well. If the 2D spatial locations are replaced by two other attributes in a corresponding attribute space, then an attribute trajectory can be generated. The definition of attribute trajectory is given in Definition 4.

**Definition** **4** **(Attribute** **trajectory).** *Given an object O, assume its bivariate time series is* $TS_{1}=\left\{\left(u_{1},t_{1}\right),\left(u_{2},t_{2}\right),\ldots ,\left(u_{n},t_{n}\right)\right\}$ *and* $TS_{2}=\left\{\left(v_{1},t_{1}\right),\left(v_{2},t_{2}\right),\ldots ,\left(v_{n},t_{n}\right)\right\}$ *, then* $AttrTraj=\left\{\left(u_{1},v_{1},t_{1}\right),\left(u_{2},v_{2},t_{2}\right),\ldots ,\left(u_{n},v_{n},t_{n}\right)\right\}$ *is considered as an attribute trajectory.*

**Figure 2:**
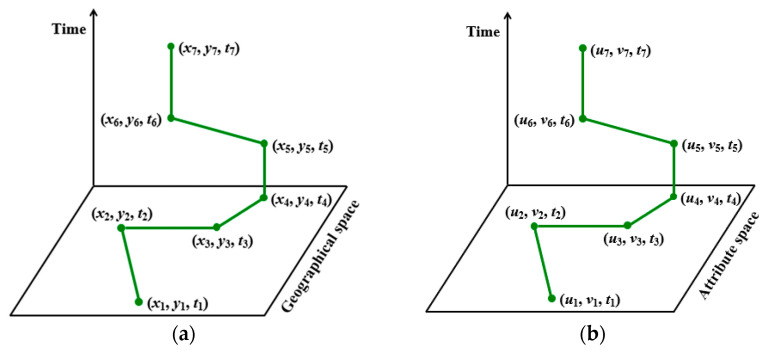
Illustration of traditional trajectory and attribute trajectory: (**a**) a traditional trajectory, and (**b**) a novel attribute trajectory.

An illustration of trajectory and attribute trajectory is shown in Figure 2. Note that Figure 2a illustrates a traditional trajectory, and Figure 2b illustrates a novel attribute trajectory. According to Figure 2, we can observe that one of the most significant differences between the two kinds of trajectories is the plane space in the coordinate systems: for the traditional trajectory, its plane space is usually a geographical space, but for the novel attribute trajectory, its coordinate system is generally an attribute space. In the attribute coordinate system, each of the two axes denotes a corresponding attribute. Given the high similarity in the structures of a traditional trajectory and an attribute trajectory, traditional trajectory mining techniques can be adapted to mine information for attribute trajectory.

#### 2.1.3. Geographical Flock

**Definition** **5** **(Flock).** *Given a set of n trajectories of n moving objects, an (r, m, k)-flock F during a time interval* $I=[t_{i},t_{j}]$ *(where* j−i+1≥k *) consists of at least m objects such that for each discrete timestamp* $t_{i}$ *there exists a disk of radius r containing all m objects.*

**Definition** **6** **(Geographical** **flock).** *Given a set of n attribute trajectories of n geographical units, an (r, m, k)-geographical flock GF during a time interval* $I=[t_{i},t_{j}]$* (where *j−i+1≥k*) consists of at least m geographical units such that for each discrete timestamp *$t_{i}$* there exists a disk of radius r containing all m attribute values of geographical units.*

**Figure 3:**
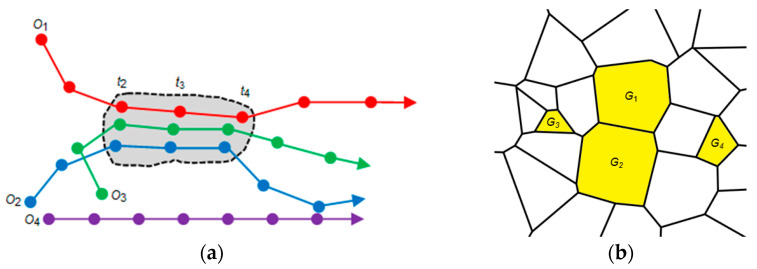
Illustration of flock and geographical flock: (**a**) a flock, and (**b**) a geographical flock.

Figure 3 gives an illustration of flock and geographical flock. Figure 3a illustrates a flock, wherein three objects (i.e., *O*_1_, *O*_2_ and *O*_3_) form a flock during the time period from *t*_2_ to *t*_4_, and Figure 3b illustrates a geographical flock, which indicates that four geographical units (i.e., *G*_1_, *G*_2_, *G*_3_ and *G*_4_) can form a geographical flock (assuming their attribute values at the same timestamp during a time duration are within a given threshold). To assist understanding, a geographical flock can be informally understood as “a group of geographical units with similar attribute values lasting for a specific time duration.”

### 2.2. Study Area and Data

**Figure 4:**
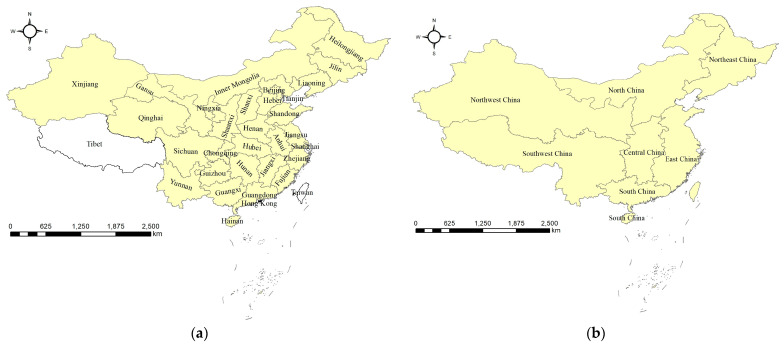
The visualization of the study area: (**a**) the 30 provinces, and (**b**) the seven geographical regions.

China includes 23 provinces, five autonomous regions, four municipalities, and two special administrative regions. Due to the problem of missing data from the provinces of Tibet, Taiwan, Hong Kong (special administrative region) and Macao (special administrative region), we take only the other remaining provinces/autonomous regions/municipalities as the study area. To facilitate interpretation, we group all of these under the term “provinces”. Therefore, 30 provinces are involved in the study area. As we aim to discover geographical flock patterns to support the coordinated formulation of potential policies in controlling carbon emissions, we also conduct our case study on the level of geographical region, which includes seven altogether. The detailed information of the 30 provinces and the seven geographical regions are listed in Table 1, in which the names and IDs of all the provinces and geographical regions are included. The visualization of the study area is shown in Figure 4, in which Figure 4a denotes the 30 provinces and Figure 4b the seven geographical regions. Note that in Figure 4a, the white color indicates that the corresponding province is not included in the study area.

The data used in this study were acquired from the Carbon Emission Accounts and Datasets for emerging economies (CEADs) [36], which provides datasets related to carbon emissions in China at either province level, prefecture-level city level, or county level with different time span (year as the unit) to the public. The data of province-level CO_2_ emissions adopted in this study include the exact information of CO_2_ emissions of the 30 provinces. The time span of the data is from 1998 to 2019, and the temporal resolution is one year. Specifically, the data contain the sectoral CO_2_ emissions inventory for all 30 provinces. We use the total consumption of all sectoral CO_2_ emissions inventory as the value of the total CO_2_ emissions for each province. Note that we adopt the linear interpolation methods to derive the corresponding values for the two missing data. Two important attributes, i.e., the total CO_2_ emissions per year and the growth rate of total CO_2_ emissions per year, which are studied in a previous work [37], are used to generate the corresponding attribute trajectory of each province and geographical region. 

### 2.3. Methodology

We develop an STG-based approach to discover geographical flock patterns from bivariate time series data (e.g., CO_2_ emission data). The methodology includes three main parts: first, the CO_2_ emission data are transformed to attribute trajectory data; second, the STGs are generated from attribute trajectory data; third, specific types of geographical flock patterns are discovered from the generated STGs. In the following, each part will be described in more detail.

#### 2.3.1. Generating Attribute Trajectory Data

As introduced in Definition 2, a bivariate time series has two different attributes. The core of generating a corresponding attribute trajectory from a bivariate time series can be seen in Definition 4. Note that to make effective comparisons between the values of the two attributes (i.e., the total CO_2_ emission, and the growth rate of total CO_2_ emission), it is necessary to consider a normalization operation. The adopted normalization method is the Z-Score method, which is represented in Equation (1):

(1) $$v\_norm_{i}=\frac{v_{i}-E}{\sigma }$$

where v_norm is the normalized value of the original attribute value *v*, and *E* and *σ* are the mean value and the standard deviation of all the original attribute values, respectively. According to this method, the attribute trajectory data of all geographical units (i.e., provinces and geographical regions) can be fully generated.

#### 2.3.2. Generating STGs

The STGs are generated based on regularly sampled attribute trajectory data. Similar to other types of graphs, a STG can also be represented as G={V, E}, where $V=\left\{v_{1},\;v_{2},\;\ldots ,\;v_{n}\right\}$ and $E=\left\{v_{i}v_{j}\}(1\leq i\leq n,\;1\leq j\leq n\right)$ denote the set of vertices and edges, respectively. Two steps are required to construct an STG: the generation of vertices and the construction of edges.

As mentioned in Definition 6, three essential parameters (i.e., *r*, *m* and *k*) are required in a geographical flock. The parameter *r* is considered when generating vertices. If the “locations” of geographical units in the attribute space at the same timestamp are within a circle of radius *r* whose value is user-defined, then the corresponding geographical units are considered to be involved in the same vertex. It should be noted that the selection of the base geographical unit (i.e., the geographical unit whose location at a timestamp is regarded as the center of the corresponding circle) is important. In this approach, we propose that for each timestamp, the geographical unit whose distance in the attribute space is closest to the centroid of all geographical units is considered as the base geographical unit. In this way, the vertices at each timestamp can be automatically generated. When constructing the edges, two basic principles are adopted: (1) any two vertices at the same timestamp are not allowed to be connected, and (2) any two vertices at two consecutive timestamps have to be connected if at least one common geographical unit is involved in both vertices. Thus, the edges can be fully constructed.

#### 2.3.3. Discovering Specific Types of Geographical Flock Patterns

Once the STGs of the geographical units are generated, the geographical flock patterns can then be discovered based on the STGs. Before the discovery operation, the STGs whose time durations are less than a user-defined value of *k* have to be deleted. For the remaining STGs, an iteration method is adopted to find all groups of geographical units, including the IDs of geographical units and the corresponding time interval in each group. Note that for each group, the number of involved geographical units has to be at least *m* (which is user-defined) and the time duration has to be at least *k*. Thus, the groups of geographical units which meet the above conditions are considered to be geographical flocks. A geographical flock is represented as {*IDs of geographical units*}|[*start time*, *end time*]. For example, the geographical flock {1, 2, 3}|[0, 5] indicates that it includes three geographical units whose IDs are 1, 2 and 3, respectively, and lasts for six continuous timestamps which are 0, 1, 2, 3, 4 and 5, respectively.

The geographical flock patterns can be further classified into different types. Based on more refined information, specific types of geographical flock patterns can be derived, according to which more elaborate insights may be provided in practice. In this paper, we propose to use two specific criteria to derive various types of geographical flock patterns, i.e., the high–low attribute values criterion, and the extreme number–duration values criterion. In detail, the high–low attribute values criterion is adopted to derive types of geographical flocks whose attribute values meet certain conditions (e.g., higher or lower than a pre-defined threshold represented by the corresponding parameter of *high_threshold* and *low_threshold*), and the extreme number–duration values criterion is utilized to derive types of geographical flocks whose number of members (i.e., geographical units) and time duration have extreme values (e.g., maximum/minimum/longest/shortest). As for the high–low attribute values criterion, since there are two attributes, and the value of each attribute may be higher than or lower than a threshold, four (i.e., 2 × 2) specific types of geographical flock patterns can be derived. For the extreme number–duration values criterion, the extreme number can be either maximum or minimum, and the extreme duration can be either longest or shortest, thus, four (i.e., 2 × 2) specific types of geographical flock patterns can be derived in either case. The derived types of geographical flock patterns are listed in detail in Table 2. To avoid confusion, we use two different encoding methods (i.e., A, B, C, D and I, II, III, IV) to distinguish the types of geographical flock patterns, and each type of geographical flock pattern is represented by a corresponding type ID. 

For types A, B, C and D, a threshold is defined to distinguish the cases of high and low values. We take the percentile method, in which we only give a desired percentile value for distinguishing the high case and the low case, so that the real attribute values for the corresponding high case and low case can be automatically determined. The real attribute values are considered the thresholds. This can effectively avoid the difficulty of giving real attribute values as thresholds in reality. Based on this, the corresponding types of geographical flock patterns meeting the conditions of high and low cases can be extracted. For types I, II, III and IV, we first calculate the number of members and time duration for each discovered geographical flock, we then find the maximum value and minimum value for the number of members and time duration, respectively, and finally extract the geographical flocks meeting the corresponding conditions. Thus, all the specific types of geographical flock patterns can be extracted. Nevertheless, in reality it cannot be guaranteed that all specific types can be discovered simultaneously for each criterion under the same combination of parameter values. One can select the optimal results by adjusting parameter values and considering his/her specific desires.

## 3. Results and Discussion

The results are presented on two levels: the province level and the geographical region level. As introduced in Definition 6, three essential parameters (i.e., *r*, *m* and *k*) are involved in geographical flock, and different combination of parameter values may lead to different results. Therefore, it is necessary to determine suitable parameter values when discovering geographical flock patterns. An important principle for determining suitable parameter values is that a fit number (i.e., neither too large nor too small) of geographical flocks has to be discovered. This is because, if the number of discovered geographical flocks is too large (or too small), it may provide too much (or insufficient) information, which can lead to corresponding difficulties in interpretation. Based on this, for parameter *r*, the smaller its value, the better, because a small value indicates that the changes of attributes are in a small fluctuation range; for parameters *m* and *k*, larger values are preferred, because large values indicate that the geographical flock patterns that are more meaningful may be discovered. By considering the strategies for selecting potential parameter values mentioned above, we have tested a large number of different combinations of parameter values. Due to space limitations, only the significant geographical flock patterns from our perspective are presented in detail in the following.

### 3.1. Geographical Flock Patterns on the Province Level

As mentioned in Section 2.3.3, two criteria are proposed to derive specific types of geographical flock patterns, therefore, the results based on each criterion at the province level will be presented below.

#### 3.1.1. The High–Low Attribute Values

Four representative geographical flocks were discovered based on the criterion of the high–low attribute values under the combination of parameter values for *r* = 15, *m* = 3, *k* = 3, *high_threshold* = 70, *low_threshold* = 30. The detailed information of the four geographical flocks can be seen in Table 3. From Table 3 we can see that, among all the geographical flocks, one belongs to type C and the other three belong to type B, while none were discovered for types A and D. This is reasonable, because it cannot be guaranteed that all types of geographical flock patterns can be discovered simultaneously under the same combination of parameter values. 

According to the four geographical flocks, we can see that three groups of provinces had both a high amount and a low growth rate of total CO_2_ emission during three continuous years (corresponding to type B). Specifically, the three groups and the corresponding continuous years are the provinces of Zhejiang, Hunan and Guangdong from 2012 to 2014, the provinces of Shanxi, Zhejiang and Guangdong from 2013 to 2015, and the provinces of Liaoning, Zhejiang, Hubei and Sichuan from 2014 to 2016. Generally speaking, the provinces in the same geographical flock performed well in controlling the growth rate of total CO_2_ emission in the corresponding years, which indicates that the measures and policies adopted have been effective. However, in the corresponding years, their total amounts of CO_2_ emissions were still very high, which demonstrates that more effective measures may be taken to control the total amount of carbon emissions. Secondly, we can see that one group of provinces had both a low amount and a high growth rate of total CO_2_ emission (corresponding to type C). The specific provinces and the corresponding years are Hainan, Ningxia and Xinjiang from 2002 to 2004. It can be inferred that the three provinces have performed well in controlling the total amount of CO_2_ emission, but that the effects of controlling the growth rate of total CO_2_ emissions were not that satisfactory.

**Figure 5:**
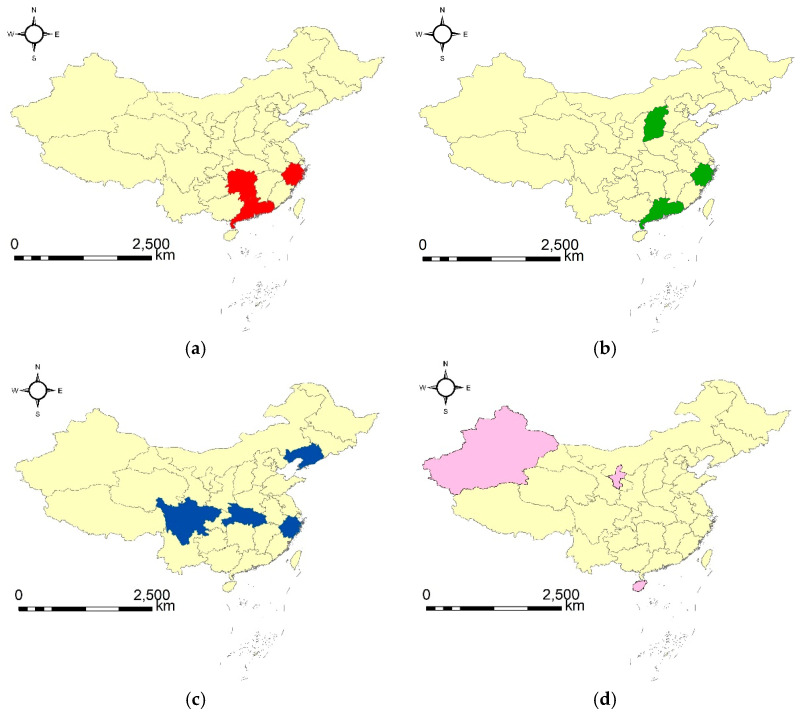
The discovered geographical flock patterns: (**a**) {11, 18, 19}|[2012, 2014] (type B), (**b**) {4, 11, 19}|[2013, 2015] (type B), (**c**) {6, 11, 17, 23}|[2014, 2016] (type B), and (**d**) {21, 29, 30}|[2002, 2004] (type C).

To further explore the spatial relations of the provinces in each geographical flock, we visualize each geographical flock on the map by setting a different color. The visualization is shown in Figure 5, in which each figure corresponds to a geographical flock, and the provinces involved in the same geographical flock are denoted the same color. From Figure 5, we can see that there appears to be a stronger spatial relation for the provinces in the geographical flock type B (Figure 5a–c) than type C (Figure 5d), as the provinces involved in the geographical flock type B (Figure 5a–c) are relatively close to each other in space, while the provinces involved in the geographical flock type C (Figure 5d) have a relatively further geographical distance from each other. Additionally, an interesting finding is that the provinces involved in type B (Figure 5a–c) have generally stronger comprehensive strength than those involved in type C (Figure 5d). This indicates that the related factors (such as economy, population and industry) of the provinces in the same type of geographical flock pattern may be similar, an obvious difference in the related factors may exist in the provinces involved in different types of geographical flock patterns. However, this still needs further exploration. In summary, the geographical flock patterns discovered based on this criterion reveal several interesting findings, which can be fully considered when conducting inter-provincial collaborations and when making coordinated policies to effectively control the amount and growth rate of carbon emissions.

#### 3.1.2. The Extreme Number–Duration Values

Two significant geographical flocks were discovered based on the criterion of the extreme number–duration values under the combination of parameter values for *r* = 10, *m* = 5, and *k* = 3. The full information of the two geographical flocks is shown in Table 4, from which we can observe that one belongs to type II and the other belongs to type III. As for types I and IV, none has ever been discovered under this specific combination of parameter values. 

The results show that a maximum number of ten provinces have had similar evolution patterns in both the amount and the growth rate of total CO_2_ emission in a shortest duration of three continuous years. The specific provinces and the corresponding years are Beijing, Shanxi, Zhejiang, Anhui, Jiangxi, Henan, Hubei, Guangxi, Gansu and Xinjiang from 1998 to 2000. This demonstrates that the ten provinces formed a maximum group which had similar CO_2_ emissions and lasted for three continuous years. This finding would be applicable to meet the needs of detecting the largest number of provinces with similar CO_2_ emission so that closer inter-provincial cooperation may be carried out. Secondly, a minimum number of five provinces had similar evolution patterns in both the amount and the growth rate of total CO_2_ emission in a maximum duration of four continuous years. The specific provinces and the corresponding years are Heilongjiang, Zhejiang, Anhui, Hubei and Sichuan from 2014 to 2017. This shows that the five provinces have had a similar evolution pattern of CO_2_ emission in a maximum duration of four years. Therefore, if one would like to know the provinces which lasted for the longest duration, this finding can provide the ideal answer. In our view, the results can provide useful suggestions to related governmental departments. Furthermore, one can detect the very groups of provinces which have had similar evolution patterns in carbon emissions by adjusting the values of the three parameters to meet his/her specific demands.

**Figure 6:**
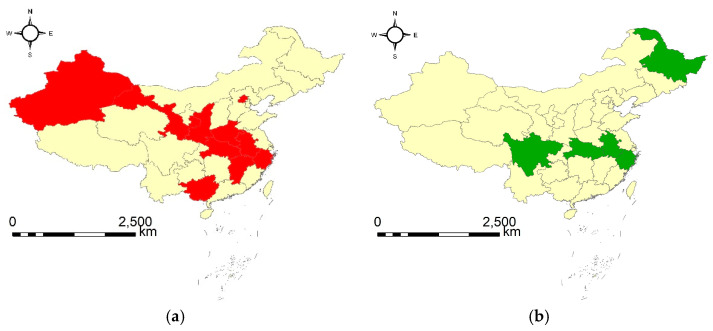
The discovered geographical flock patterns: (**a**) {1, 4, 11, 12, 14, 16, 17, 20, 27, 30}|[1998, 2000] (type II), and (**b**) {8, 11, 12, 17, 23}|[2014, 2017] (type III).

The visualization of the two geographical flocks can be seen in Figure 6, which gives an overview of the spatial relations of the provinces involved in the same geographical flock. From Figure 6 we can see that Figure 6a exhibits an overall strong spatial relation for all the involved provinces, and Figure 6b presents a strong spatial relation for most of the involved provinces. Therefore, we can infer that geographical locations may have strong effects on the potential groups of provinces which can form a specific type of geographical flock, but other factors can also have particular effects on the final formulation of potential geographical flocks. The findings indicate that further and finer explorations may be conducted to gain further insight on why the provinces with relatively weak spatial relations can form particular geographical flocks so that more scientific, precise and flexible policies and/or strategies can be made in the future.

### 3.2. Geographical Flock Patterns on the Geographical Region Level

The discovered geographical flock patterns based on each criterion on the geographical region level will be presented in detail in the following.

#### 3.2.1. The High–Low Attribute Values

Two significant geographical flocks were discovered under the criterion of the high–low attribute values. The exact information of the two geographical flocks is listed in Table 5. From Table 5 we can see that between the two geographical flocks, one belongs to type B and the other belongs to type C, and none have been discovered for types A and D under this specific combination of parameter values (i.e., *r* = 10, *m* = 2, *k* = 3, *low_threshold* = 40, *high_threshold* = 60). 

The two geographical flocks reveal that one group of geographical regions has had both a high amount and a low growth rate of total CO_2_ emission during three continuous years (corresponding to type B). The specific geographical regions and corresponding continuous years are Northeast China and Central China from 2015 to 2018. Secondly, one group of geographical regions has had both a low amount and a high growth rate of total CO_2_ emission (corresponding to type C). The corresponding geographical regions and continuous years are Northeast China and South China from 2005 to 2007. Note that a common geographical region involved in both groups is Northeast China. According to the results, it can be inferred that Northeast China may have taken effective measures in controlling the growth rate of CO_2_ emissions, as it has been keeping a steady low growth rate in recent years (2015 ~ 2018) while the growth rate was relatively high in years prior to that (2005 ~ 2007). Based on the results, potential suggestions may be provided to related national governmental departments to carry out effective regional cooperation to work out more targeted policies in better controlling carbon emissions. 

**Figure 7:**
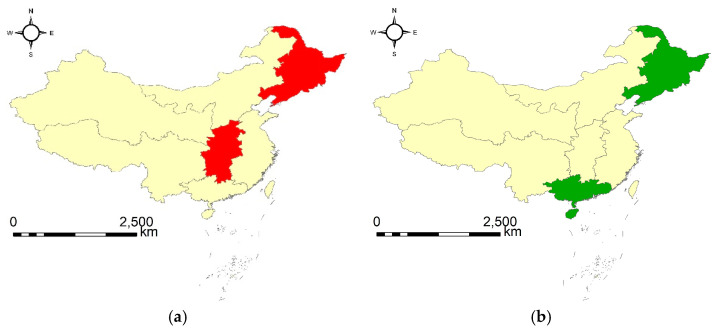
The discovered geographical flock patterns: (**a**) {2, 4}|[2015, 2018] (type B), and (**b**) {2, 5}|[2005, 2007] (type C).

Figure 7 exhibits the visualization of the two geographical flocks. From Figure 7 we can see that the geographical regions in geographical flock type B (Figure 7a) have relatively stronger spatial relations than those in type C (Figure 7b), which coincides well with the corresponding finding in Section 3.1.1. This may provide us additional useful clues on how to produce more scientific strategies to better control carbon emissions in the future.

#### 3.2.2. The Extreme Number–Duration Values

Three typical geographical flocks were discovered under the criterion of the extreme number–duration values. The specific information of all the geographical flocks can be seen in Table 6, from which we can see that, among the three geographical flocks, two belong to both type I and type III, and the other belongs to both type II and type IV. An obvious difference of these results from the previous results is that all types of geographical flock patterns have been discovered under this specific combination of parameter values (i.e., *r* = 5, *m* = 2, *k* = 3).

The main findings based on the three geographical flocks can be summarized as follows. (1) Two groups of geographical regions have had similar evolution patterns in both the amount and the growth rate of total CO_2_ emission in a maximum duration of four continuous years. The number of geographical regions involved in each group is two, which is both the maximum (corresponding to type I) and the minimum (corresponding to type III) among all the discovered geographical flock patterns; the corresponding geographical regions and continuous years are Northeast China and South China from 2005 to 2008, and Northeast China and Southwest China from 2013 to 2016, respectively. (2) One group of geographical regions has had similar evolution patterns in both the amount and the growth rate of total CO_2_ emission in a minimum duration of three continuous years. Similar to the other two geographical flocks, the number of geographical regions involved in this geographical flock is both the maximum and the minimum, thus it belongs to both type II and type IV. The detailed information of this geographical flock is the geographical regions of Central China and Northwest China from 2014 to 2016. According to the results, the abovementioned geographical regions involved in the same geographical flock may carry out closer cooperation to determine more scientific measures to better control and release the emissions of carbons. For example, Northeast China may corporate closely with other regions, such as South China and Southwest China, to explore the specific reasons why similar emission patterns have appeared.

**Figure 8:**
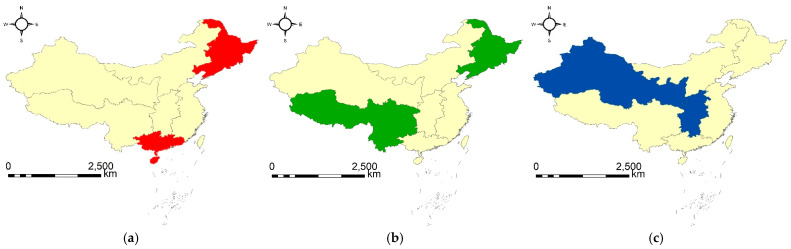
The discovered geographical flock patterns: (**a**) {2, 5}|[2005, 2008] (type I, III), (**b**) {2, 6}|[2013, 2016] (type I, III), and (**c**) {4, 7}|[2014, 2016] (type II, IV).

The corresponding visualization of the three geographical flocks is shown in Figure 8, from which the spatial distribution of the geographical regions involved in the same geographical flock can be clearly seen. An interesting finding is that the geographical flock of type II/IV (Figure 8c) exhibits strong spatial relations, while the spatial relations of the geographical flock of type I/III (Figure 8a,b) appear relatively weak. By considering the similar findings in spatial relations in Section 3.1.2, related departments may gain additional insights to propose more elaborate plans so that carbon emissions can be controlled in a more scientific way in the future.

## 4. Conclusions

Climate change has become one of the greatest global challenges, and one which can greatly affect humankind. A significant solution for mitigating climate change is to reduce the emissions of greenhouse gas, particularly CO_2_. To better support the establishment of effective policies for reducing CO_2_, it is crucial to consider specific sorts of important emission patterns that exist between provinces and/or geographical regions. This paper takes geographical flock patterns as the very important kind of emission pattern which deserves further investigation. We propose an STG-based approach to effectively discover geographical flock patterns. The approach mainly includes three steps, i.e., generating attribute trajectories from CO_2_ emission data, generating STGs from attribute trajectories, and discovering specific types of geographical flock patterns. In general, eight different types of geographical flock patterns are derived based on two different criteria (i.e., the high–low attribute values criterion and the extreme number–duration values criterion). A case study was conducted on two levels, i.e., the province level and the geographical region level, based on CO_2_ emission data in China. The results of the case study demonstrate that the proposed approach is effective in discovering the different types of geographical flock patterns, and potentially useful suggestions and insights can be provided to related departments to assist in policy making and in the coordinated control of carbon emissions in the future.

Given the importance of investigating the evolution patterns of CO_2_ emission between different geographical units, we only took the province-level carbon emission data for case studies. Although the results based on the province-level CO_2_ emission data appear effective, finer results are still needed. Therefore, fine-granularity CO_2_ emission data (e.g., data at the city-level) can be adopted as new datasets for further studies to obtain more precise insight. In addition, other attributes which are meaningful to carbon emission can be used to generate attribute trajectories so that the insights and findings can be further extended. Furthermore, new criteria can be developed and adopted to derive specific types of geographical flock patterns according to one’s specific desire, according to which new findings might be acquired.

## Figures and Tables

**Table 1:** The detailed information of the provinces and geographical regions included in the study area.

Province	ID	Geographical Region	ID
Beijing	1	North China	1
Tianjin	2		
Hebei	3		
Shanxi	4		
Inner Mongolia	5		
Liaoning	6	Northeast China	2
Jilin	7		
Heilongjiang	8		
Shanghai	9	East China	3
Jiangsu	10		
Zhejiang	11		
Anhui	12		
Fujian	13		
Jiangxi	14		
Shandong	15		
Henan	16	Central China	4
Hubei	17		
Hunan	18		
Guangdong	19	South China	5
Guangxi	20		
Hainan	21		
Chongqing	22	Southwest China	6
Sichuan	23		
Guizhou	24		
Yunnan	25		
Shaanxi	26	Northwest China	7
Gansu	27		
Qinghai	28		
Ningxia	29		
Xinjiang	30		

**Table 2:** The specific types of geographical flock patterns.

Criterion	Explanation	Type ID
The high–low attribute values	High *X* attribute value and high *Y* attribute value	A
	High *X* attribute value and low *Y* attribute value	B
	Low *X* attribute value and high *Y* attribute value	C
	Low *X* attribute value and low *Y* attribute value	D
The extreme number–duration values	Maximum number and longest duration	I
	Maximum number and shortest duration	II
	Minimum number and longest duration	III
	Minimum number and shortest duration	IV

**Table 3:** The discovered geographical flock patterns based on the criterion of the high–low attribute values on the province level.

ID	Geographical Flock	Corresponding Provinces	Type ID
1	{11, 18, 19}|[2012, 2014]	Zhejiang, Hunan, Guangdong	B
2	{4, 11, 19}|[2013, 2015]	Shanxi, Zhejiang, Guangdong	B
3	{6, 11, 17, 23}|[2014, 2016]	Liaoning, Zhejiang, Hubei, Sichuan	B
4	{21, 29, 30}|[2002, 2004]	Hainan, Ningxia, Xinjiang	C

**Table 4:** The discovered geographical flock patterns based on the criterion of the extreme number-duration values on the province level.

ID	Geographical Flock	Corresponding Provinces	Type ID
1	{1, 4, 11, 12, 14, 16, 17, 20, 27, 30}|[1998, 2000]	Beijing, Shanxi, Zhejiang, Anhui, Jiangxi, Henan, Hubei, Guangxi, Gansu, Xinjiang	II
2	{8, 11, 12, 17, 23}|[2014, 2017]	Heilongjiang, Zhejiang, Anhui, Hubei, Sichuan	III

**Table 5:** The discovered geographical flock patterns based on the criterion of the high–low attribute values on the geographical region level.

ID	Geographical Flock	Corresponding Provinces	Type ID
1	{2, 4}|[2015, 2018]	Northeast China, Central China	B
2	{2, 5}|[2005, 2007]	Northeast China, South China	C

**Table 6:** The discovered geographical flock patterns based on the criterion of the extreme number-duration values on the geographical region level.

ID	Geographical Flock	Corresponding Provinces	Type ID
1	{2, 5}|[2005, 2008]	Northeast China, South China	I, III
2	{2, 6}|[2013, 2016]	Northeast China, Southwest China	I, III
3	{4, 7}|[2014, 2016]	Central China, Northwest China	II, IV

## Data Availability

Data will be made available on request.
